# Renal cell carcinoma with venous extension: prediction of inferior vena cava wall invasion by MRI

**DOI:** 10.1186/s40644-018-0150-z

**Published:** 2018-05-03

**Authors:** Lisa C. Adams, Bernhard Ralla, Yi-Na Y. Bender, Keno Bressem, Bernd Hamm, Jonas Busch, Florian Fuller, Marcus R. Makowski

**Affiliations:** 1 Department of Radiology, Charité, Charitéplatz 1, 10117 Berlin, Germany; 2 Department of Urology, Charité, Charitéplatz 1, 10117 Berlin, Germany; 3 Department of Urology, Charité, Hindenburgdamm 30, 12200 Berlin, Germany

**Keywords:** Renal cell carcinoma, Inferior vena cava thrombus, Magnetic resonance imaging, Preoperative planning, Sensitivity and specificity

## Abstract

**Background:**

Renal cell carcinoma (RCC) are accompanied by inferior vena cava (IVC) thrombus in up to 10% of the cases, with surgical resection remaining the only curative option. In case of IVC wall invasion, the operative procedure is more challenging and may even require IVC resection. This study aims to determine the diagnostic performance of contrast-enhanced magnetic resonance imaging (MRI) for the assessment of wall invasion by IVC thrombus in patients with RCC, validated with intraoperative findings.

**Methods:**

Data were collected on 81 patients with RCC and IVC thrombus, who received a radical nephrectomy and vena cava thrombectomy between February 2008 and November 2017. Forty eight patients met the inclusion criteria. Sensitivity and specificity as well as the positive and negative predictive values were calculated for preoperative MRI, based on the assessments of the two readers for visual wall invasion. Furthermore, a logistic regression model was used to determine if there was an association between intraoperative wall adherence and IVC diameter.

**Results:**

Complete occlusion of the IVC lumen or vessel breach could reliably assess IVC wall invasion with a sensitivity of 92.3% (95%-CI: 0.75–0.99) and a specificity of 86.4% (95%-CI: 0.65–0.97) (Fisher-test: *p*-value< 0.001). The positive predictive value (PPV) was 88.9% (95%-CI: 0.71–0.98) and the negative predictive value reached 90.5% (95%-CI: 0.70–0.99). There was an excellent interobserver agreement for determining IVC wall invasion with a kappa coefficient of 0.90 (95%CI: 0.79–1.00).

**Conclusions:**

The present study indicates that standard preoperative MR imaging can be used to reliably assess IVC wall invasion, evaluating morphologic features such as the complete occlusion of the IVC lumen or vessel breach. Increases in IVC diameter are associated with a higher probability of IVC wall invasion.

## Background

Renal cell carcinoma (RCC) represent approximately 2–3% of all tumors and show a propensity for vascular growth with up to 10% of patients developing an inferior vena cava (IVC) thrombus [1, 2]. IVC wall invasion is a negative prognostic factor [3], whereby positive renal or caval vein margins are associated with worse survival outcomes [4, 5]. To date, surgical resection remains the only curative option, offering a 5-year-survival of up to 40–65% for RCC with intravascular growth, which is reduced in cases with IVC wall invasion [6–8].

In case of IVC wall invasion, surgery is more challenging, because it may necessitate segmental resection or even prosthetic replacement to prevent postoperative recurrence or venous insufficiency [9, 10]. The need for segmental resection or prosthetic replacement is typically determined intraoperatively. Therefore, the ability to predict IVC invasion preoperatively would be a clear advantage in terms of preoperative planning and a priori patient information.

High quality diagnostic imaging is a cornerstone of preoperative planning and management. With regard to the presence and extent of IVC invasion, MR is a powerful and accurate tool and is suggested to be more reliable than computed tomography (CT) [11]. However, data on the prediction of venous wall invasion by preoperative imaging are sparse. There have been only a limited number of studies - most of them with older generation MR scanners and a small number of patients - investigating the ability of CT or MRI to assess the extent of wall invasion and vena caval tumor extension [7, 9, 12–14]. While breach of the vessel wall with tumor signal on both sides of the vessel wall has been demonstrated to be a reliable sign of IVC wall invasion [9, 15], contact of the IVC thrombus with the vessel wall could not be established as a reliable predictor so far.

## Methods

In the present study, we aimed to evaluate the accuracy of preoperative standard MRI for determining or ruling out wall invasion of the IVC, based on morphologic features such as vessel wall contact or vessel wall breach, with imaging findings being validated with intraoperative results. Furthermore, we sought to test for the potential association between wall invasion and IVC diameter or thrombus enhancement.

### Study design and population

This retrospective study was approved by the Institutional Review Board. Between February 2008 and November 2017, 81 patients with histologically proven RCC and IVC thrombus received a radical nephrectomy and vena cava thrombectomy with an intraoperative assessment of IVC wall invasion. Of these patients, 48 patients obtained a preoperative MRI examination with a clinical routine protocol at a 1.5 T unit and could be included in our analysis, aiming to validate in vivo findings of IVC wall invasion with intraoperative findings.

The patient sample consisted of a total of 48 patients (10 women and 38 men, aged 38–79, mean 64.9 ± 9.8). The median time between the preoperative imaging and the date of surgery was 16.1 (± 13.3) days. With regard to the composition of the thrombus, there were 8 patients with bland thrombus (0 cases IVC wall invasion), 19 patients with tumor thrombus (16 cases with wall invasion) and 21 patients with mixed content (10 cases with wall invasion), whereby mixed content refers to a coexistence of bland thrombus and tumor thrombus. Circumferential cavectomy with prosthetic replacement of the IVC was performed in only 3 of the 48 patients (6.3%), whereas the other patients received a reconfiguration with continuous suturing. In the 3 cases with circumferential cavectomy, IVC tumor invasion was histologically confirmed. In 9 of the 48 patients (18.8%) with level IV thrombi, a cardiopulmonary bypass had to be used. During histological examination, 40 of the patients were revealed to have a clear cell RCC, seven patients showed papillary carcinomas and one patients had an undifferentiated renal carcinoma, which could not be clearly classified. An overview of the patients’ characteristics is provided by Table 1.

**Table 1:** Characteristics of the Study Population

Number of patients	48
Number of men/women	38/10
Mean age at surgery (range; SD)	64.9 (38–79; 9.8)
Involvement of the right kidney (number)	37
Thrombus level (number, %)	
I	9 (18.8)
II	17 (35.4)
III	13 (27.1)
IV	9 (18.8)
Fuhrman grade (number, %)	
1	2 (4.3)
2	19 (40.4)
3	18 (38.3)
4	8 (17.0)
TNM classification (number, %)	
T1	1 (2.1)
T2	1 (2.1)
T3a	15 (31.2)
T3b	22 (45.8)
T3c	7 (14.6)
T4	2 (4.2)
Number of clear cell carcinoma (%)	40 (83.3)
Number of papillary carcinoma (%)	7 (14.6)
Presence of preoperative metastases (number, %)	16 (33.3)

### Imaging protocol

The MRI examinations from our hospital were performed on 1.5 T units (Aera/Avanto/Symphony/Sonata, Siemens Medical Solutions, Erlangen, Germany) with dedicated body-phased-array coils. All patients underwent a clinical routine imaging of the kidneys at 1.5 T, which included transverse, coronal and sagittal T2 half Fourier single-shot turbo spin echo sequences (HASTE), unenhanced axial 3D gradient echo pulse T1-weighted (FLASH) images, a T1- FLASH angiography, obtained prior to and after the intravenous administration of contrast agents, and a fat saturated volumetric interpolated breath-hold examination (VIBE) T1 3D sequence (see Table 2 for tabulated magnetic resonance imaging parameters).

**Table 2:** Tabulated imaging parameters of the magnetic resonance sequences

Type of acquisition	T2 HASTE^a^ axial	T2 HASTE^a^ coronary	T2 TSE^b^ axial (PACE)^b^	T1 FLASH^c^	Angiography T1 FLASH^c^	T1 VIBE^d^
Repetition time, TR (ms)	800	800	2430	186	2.88	4.74
Echo time, TE (ms)	94	89	79	4.76	0.98	2.38
Field of view (FOV)	340 × 340	400 × 400	340 × 340	340 × 340	500 × 500	373 × 373
Matrix size	320 × 320	320 × 320	320 × 320	320 × 320	512 × 512	320 × 320
Slice thickness (mm)	5	5	4	4	1.4	3
Pixel bandwidth (Hz/pixel)	300	422	260	260	440	400
Acquisition mode	2D	2D	2D	2D	3D	3D
Flip angle (°)	180	170	180	70	25	10
Voxel size	1.3 × 1.1 × 4.0	1.7 × 1.3 × 5.0	1.5 × 1.1 × 4.0	1.4 × 1.1 × 4.0	1.6 × 1.0 × 1.4	1.7 × 1.2 × 3.0

^a^Half Fourier Single-shot Turbo-spin Echo sequence

^b^Turbo Spin Echo with Prospective Acquisition Correction

^c^Fast low-angle shot magnetic resonance imaging

^d^Volumetric Interpolated Breath-hold Examination

### Level of IVC extent, tumor thrombus enhancement and IVC diameter

Level of IVC extent was stratified following the classification of tumor thrombus level according to the Mayo staging system [16]: Level I refers to thrombi that extend into the IVC to no more than 2 cm above the renal vein. Level II represents IVC thrombi extending into into the IVC to more than 2 cm above the renal vein but not to the hepatic vein, whereby Levels I and II make up for approximately 50% of the thrombi. Level III IVC thrombi are defined as extending above the hepatic veins, but below the diaphragm, making up for about 40%. Level IV IVC thrombi extend above the diaphragm or into the right atrium and represent approximately 10% [17]. IVC thrombus levels for all patients were assessed at two time points, first, during preoperative imaging, and second by exploration during surgery.

With regard to the composition of IVC thrombus, an image-based differentiation was performed for bland thrombus, which was diagnosed, when there was no thrombus enhancement, tumor thrombus, which was assumed when the signal intensity was similar to that of the RCC and mixed content, which included both features and could e.g. also refer to a tumor thrombus covered by clot.

The maximum IVC diameters were measured on an axial section in two directions, which were perpendicular to each other.

### Image analysis

All of the images were analyzed by use of PACS workstations (Centricity Radiology; GE Healthcare). All MRI images were evaluated by two radiologists blinded to the surgical and pathological findings and to the observations of the other in randomized order and in two different reading sessions, which were separated by a period of 2 weeks. IVC thrombi were analyzed based on the following properties: upper extent (infrahepatic, intrahepatic, infra-diaphragmatic or supradiaphragmatic), thrombus enhancement, IVC diameter and wall invasion. If the IVC thrombus could clearly be delineated from the vessel wall and if there was no thickening or altered signal of the low-intensity vessel wall, invasion was assumed to be absent. If there was a contact of the IVC thrombus with or even a visual breach of the IVC wall, IVC wall invasion was assumed to be present. More specifically, contact to the vessel wall referred to a loss of delineation between thrombus and vessel wall with complete occlusion of the vessel and blood signal loss in the affected area. Consequently, a diagnosis of wall invasion was made, when the tumor showed contact with the vessel wall or, if there was a breach or extension through the vessel wall [9]. Agreement between the two observers was also assessed. Finally, imaging findings were validated with intraoperative findings.

### Intraoperative evaluation and procedure

Intraoperatively, wall invasion was reported, if the IVC thrombus showed any adherence to the IVC wall. Absence of IVC wall invasion was confirmed, if the caval thrombus could be easily removed.

If the IVC does not show any signs of advanced invasion intraoperatively and there is no evidence of the resection compromising the IVC lumen, the standard operative procedure at our institution involves a combination of thrombectomy with subsequent cavorrhaphy, using continuous polypropylene suturing. If there is advanced tumorous invasion with a breach of the vessel wall IVC resection, either segmental or circumferential, become necessary.

### Statistical analysis

Statistical analysis was performed with “R” Statistical Software (Version 3.2.2, R Development Core Team, 2015). Variables were expressed as means ± standard deviations. Sensitivity and specificity as well as the positive and negative predictive values were calculated based on the assessments of the two readers for visual wall invasion. In case of a differing assessment, the opposite of the reference standard (intraoperative finding) was assumed in order to avoid an overestimation of the diagnostic performance. Cohen’s kappa coefficient was used to measure interobserver agreement for categorical variables (invasion/no invasion). The intraclass coefficient (ICC) was used to assess interobserver and intermodality reliability for continuous data. Interobserver and intermodality reliability was considered poor for ICC/kappa values less than 0.40, fair for values from 0.40–0.59, good for values from 0.60–0.74 and excellent for values above 0.75. Furthermore, a logistic regression model was used to determine if there was an association between intraoperative wall adherence and IVC diameter. Fisher’s exact test was used to assess if thrombus enhancement showed a significant association with IVC wall invasion. A *p*-value < 0.05 was considered statistically significant.

## Results

All of the 48 patients underwent extended nephrectomy and thrombectomy, with the information available from surgery being used to confirm IVC wall invasion.

### Validation with intraoperative findings

We found that contact of the IVC thrombus to or breach of the vessel wall could reliably diagnose wall invasion in preoperative MRI imaging with a sensitivity of 92.3% (95%-CI: 0.75–0.99) and a specificity of 86.4% (95%-CI: 0.65–0.97) (Fisher test: p-value < 0.001). The positive predictive value (PPV) was 88.9% (95%-CI: 0.71–0.98) and the negative predictive value reached 90.5% (95%-CI: 0.70–0.99) (refer to Table 3).

**Table 3:** Diagnostic performance of MRI with surgery as the reference standard

	Observer 1	Observer 2	Observer 1 and 2 combined
Sensitivity	0.92 (0.75–0.99)	0.96 (0.80–1.0)	0.92 (0.75–0.99)
Specificity	0.95 (0.77–1.0)	0.86 (0.65–0.97)	0.86 (0.65–0.97)
Negative predictive value	0.91 (0.72–0.99)	0.95 (0.75–1.0)	0.91 (0.70–0.99)
Positive predictive value	0.96 (0.80–1.0)	0.89 (0.72–0.98)	0.89 (0.71–0.98)

Point estimates and 95% confidence intervals are indicated in brackets

**Fig. 1:**
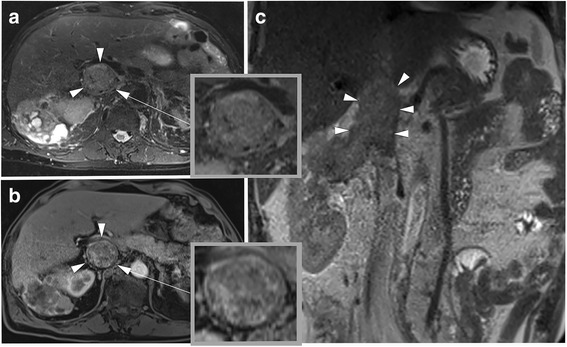
Images in a 55-year old man with a clear cell renal cell carcinoma (RCC) and an inferior vena cava (IVC) tumor thrombus with wall invasion. The RCC extends from the right kidney into the suprahepatic IVC. **a** axial fat-saturated T2-weighted image. **b** T1-weighted contrast-enhanced 3D GRE (VIBE) image (arterial phase) and (**c**), coronal T2-weighted HASTE image for anatomic reference. Note that the thrombus completely obstructs the lumen of the IVC and shows direct contact with the vessel wall (**a**, **c**). The contrast-enhanced image (**b**) demonstrates a heterogeneous enhancement of the tumor thrombus, and contact to, but no breach of the vessel wall, which makes IVC wall invasion likely. During extended nephrectomy, this thrombus was partly adherent the IVC and after extraction of the IVC thrombus, continuous suturing became necessary. VIBE = Volumetric interpolated breath-hold examination

**Fig. 2:**
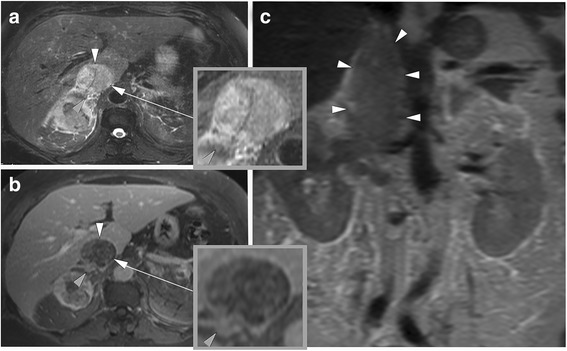
Images in a 69-year old woman with a clear cell renal cell carcinoma (RCC) and an inferior vena cava (IVC) tumor thrombus with wall invasion. The RCC extends from the right kidney into the IVC and extends into the right atrium. **a** axial fat-saturated T2-weighted image. **b** T1-weighted contrast-enhanced 3D GRE (VIBE) image contrast enhanced 3D GRE image (arterial phase) and (**c**), coronal T2-weighted HASTE image for anatomic reference. Note that the thrombus completely obstructs the lumen of the IVC, but also seems to breach the vessel wall (**a**, **c**). The contrast-enhanced image (**b**) demonstrates a heterogeneous enhancement of the tumor thrombus and a clear breach of the vessel wall (gray arrowhead), which is highly suggestive of IVC wall invasion. During extended nephrectomy, this thrombus showed strong adherence to the IVC wall and during extraction, circumferential cavectomy with vascular reconstruction became necessary. VIBE = Volumetric interpolated breath-hold examination

**Fig. 3:**
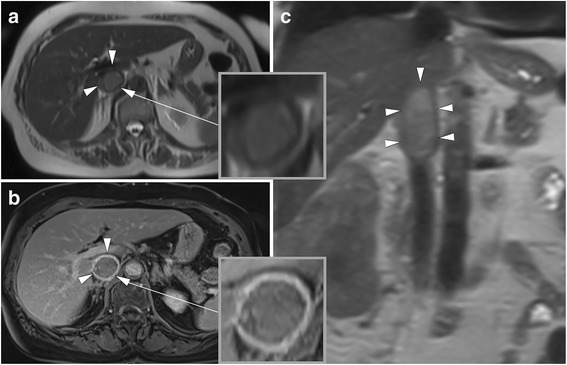
Images in a 79-year old woman with a clear cell renal cell carcinoma (RCC) and bland inferior vena cava (IVC) thrombus without wall invasion. The RCC extends from the right kidney into the infrahepatic IVC. **a** axial T2-weighted HASTE image. **b** T1-weighted contrast-enhanced 3D GRE (VIBE) image and (**c**), the coronal T2-weighted HASTE image for anatomic reference. Note that the thrombus is floating in the IVC and that there is no complete obstruction of the caval lumen (**a**, **c**). The contrast-enhanced image (**b**) demonstrates that there is no enhancement of the tumor thrombus, contact to or breach of the vessel wall, so that IVC wall invasion appears unlikely. During extended nephrectomy, this thrombus could be easily removed from the IVC without necessitating segmental resection. HASTE = Half-Fourier-acquired singe-shot turbo spin echo, VIBE = Volumetric interpolated breath-hold examination

There were 26 cases of wall invasion, of which 24 were correctly identified based on contrast-enhanced MRI (see Figs. 1 and 2 for case examples). The two patients with IVC invasion, who were considered to have a non-adherent thrombus based on MRI, were revealed to have very small areas of adherence (less than 1 cm) intraoperatively. In one of these cases, the assessment of the observers differed. Of the 22 cases, where intraoperative findings revealed no presence of wall invasion, 19 could be correctly identified with MRI (see Fig. 3 for case example). In the three cases, where MRI could not identify absence of wall invasion, this was mostly due to respiratory motion artifacts on the axial images, with the observers’ evaluation differing in two of the cases.

### Interobserver agreement for IVC wall invasion

There was an excellent interobserver agreement for determining IVC wall invasion with a kappa coefficient of 0.95 (95%CI: 0.85–0.98). In three cases, the two observers assessed the presence or absence of invasion differently, whereby the opposite of the reference standard (findings at surgery) was chosen in order to avoid an overestimation of the diagnostic performance (see Table 4).

**Table 4:** Wall invasion by inferior vena cava thrombus on MRI versus invasion determined at surgery

Observer 1 MRI	Surgery	
	Wall invasion	Absence of wall invasion
Wall invasion	24	1
Absence of wall invasion	2	21
Observer 2 MRI	Surgery	
	Wall invasion	Absence of wall invasion
Wall invasion	25	3
Absence of wall invasion	1	19
Observers 1 + 2 MRI	Surgery	
	Wall invasion	Absence of wall invasion
Wall invasion	24	3
Absence of wall invasion	2	19

In three cases, the two observers assessed the presence or absence of invasion differently. In case of a differing assessment, the opposite of the reference standard (intraoperative finding) was assumed in order to avoid an overestimation of the diagnostic performance. This combined assessment (observers 1 + 2) is shown below

Regarding IVC diameter measurements, there was also an excellent interobserver agreement with an ICC coefficient of 0.90 (95%CI: 0.79–1.00).

### Association between IVC diameter and probability of IVC invasion

Furthermore, we found that increases in IVC diameter were associated with a higher probability of IVC wall invasion, with the β-coefficient for the IVC diameter being 0.41 (standard error +/− 0.13). This influence also reached significance level (*p* = 0.015). Patients with IVC wall invasion showed a mean diameter of 42.96 mm ± 8.54 mm, while patients without wall invasion had a mean diameter 34.00 mm ± 7.25 mm. This difference was also significant (*p* = 0.0.001).

By contrast, intraoperative IVC wall invasion was not significantly related to the extent or level of IVC extent (*p* > 0.05).

### Assessment of IVC wall invasion based on MR enhancement

A clear differentiation between invasive and noninvasive IVC thrombus based on MR enhancement patterns proved to be unfeasible, as enhancement was observed in 14 out of the 22 cases without IVC invasion, indicating a high number of false positives. However, none of the non-enhancing IVC thrombi showed signs of invasion, inferring a good estimate of the false negatives. Therefore, the association between enhancement and IVC invasion was significant (Fisher test: *p* = 0.001). This matches the finding of a significant association between the composition of the IVC thrombus (if it was a bland thrombus/venous clot, a tumor thrombus or an association of both) and enhancement. While no enhancement could be observed in patients with bland tumor thrombus, there was enhancement in patients with tumor thrombus and mixed content.

## Discussion

This study suggests that preoperative MR imaging enables a reliable determination of IVC wall invasion in patients with RCC. More specifically, complete occlusion of the IVC lumen and breach of the vessel wall are indicators of IVC invasion with a high sensitivity and specificity. Furthermore, increases in IVC diameter were associated with a higher probability of IVC wall invasion.

IVC wall invasion has recently been recognized as an independent prognostic factor for the survival of patients with IVC thrombi [16, 18–20]. During surgery, IVC wall invasion requires a very challenging reconstruction beyond standard cavorrhaphy, that might include segmental resection or prosthetic replacement. However, there has been limited data with only a handful of studies and - apart from the study by Psutka et al. - with small sample sizes [9, 12, 14, 21, 22]. With regard to MR imaging, the number of studies is even more limited, as Psutka et al., for example, chose a cross-sectional approach including both MRI and CT images, with CT having a comparably lower diagnostic performance, and using a combination of multiple radiographic parameters to predict IVC invasion [14]. By contrast, we propose a purely MR-based approach with focus on the direct detection of IVC wall invasion with clearly defined morphologic parameters.

In line with our results, previous studies found tumor signal on both sides of the vessel wall to be one of the most reliable indicators of vessel breach and of IVC wall invasion [9, 12, 21]. Furthermore, Myneni et al. proposed to use the low signal intensity line of the normal vessel wall on gradient echo (GRE) and T1-weighted images as a minor criterion for vessel wall invasion [13].

In addition, contrast enhancement of the thrombus or venous wall has been suggested as a criterion for distinguishing tumor thrombus from bland thrombus and for narrowing the diagnosis to wall invasion, the theory being that the neo-vascular bed of the tumor thrombus would adhere to the venous wall, whereas the bland thrombus would not [9, 12]. In the present study, thrombus enhancement was not reliable for excluding caval wall invasion, as enhancement was observed in more than 60% of the cases, where no wall invasion was found intraoperatively. However, it proved to be reliable for excluding invasion in cases, where no IVC enhancement was observed.

Furthermore, previous studies demonstrated an association between IVC diameters and wall invasion [14, 23]. Psutka et al. considered 24 mm or more on the level of the renal vein ostium to be a probable indicator of advanced IVC invasion [14, 23]. In the present study, probability of IVC wall invasion also was higher with increasing IVC diameters, which appears logical, as bigger thrombi show a higher propensity for invasion.

The presence of IVC wall invasion has also been incorporated into the American Joint Committee on Cancer (AJCC) cancer staging criteria for RCC, changing a stage T3b to a stage T3c [24].

Previous research suggested a superior diagnostic accuracy of MRI in detecting the upper extent of IVC thrombus due to its intrinsic contrast superiority [25]. However, in more recent studies, the diagnostic performance of CT and MRI in staging the level of IVC thrombus has been regarded to be similar [26–29]. However, to our knowledge there has not been a systematic comparison between multidetector CT and MRI concerning the detection of IVC wall invasion yet.

Even though it has to be acknowledged, that the preoperative assessment of IVC invasion cannot replace the value of surgical exploration, morphologic MR features may be used to predict the risk for complicated inferior vena cava resection in a reproducible manner, which is also supported by the strong interobserver agreement we found. In clinical practice, surgeons can use the additional information to optimize their preoperative planning, e.g. in consultation with the vascular surgeons or scheduling of the most experienced surgeons. The derived information is especially important in patients, in whom reconstruction of the IVC beyond cavorrhaphy is probable, to determine the need for specific operative resources (e.g. cardiopulmonary bypass) in advance and also to individually improve prior patient information [15]. Intraoperatively, the a priori assessment of wall invasion can be a helpful adjunct to additional examinations such as duplex ultrasound or transesophageal echocardiography, which can be used to further characterize the mobility, consistency and the exact extension of the thrombus [14].

Recent studies have focused on diffusion weighted imaging (DWI) as an emerging technique for quantitative readouts, with the apparent diffusions coefficient (ADC) values representing tumor cellularity [30]. DWI has shown promise in preoperative cancer staging, e.g. for endometrial, cervical, bladder, rectal or gastric cancer [31–34]. Especially the introduction of reduced field-of-view (FOV) techniques has enabled an improved tumor delineation with higher spatial resolution, decreased partial volume averaging and less susceptibility distortion [35]. Future studies on the preoperative evaluation of IVC invasion could, therefore, include reduced FOV DWI sequences to investigate whether additional information on thrombus composition may be gained. Furthermore, functional imaging, such as dynamic contrast enhanced (DCE) MRI, may improve assessment of thrombus and wall enhancement through the quantification of contrast-enhancement characteristics and the evaluation of microcirculation parameters. During DCE imaging, several contrast enhancement parameters can be used to better differentiate tumor tissue. Previous research suggested, that especially the early postcontrast phase could be relevant for tumor detection [36]. By contrast, there were also studies suggesting, that there was limited additional value for diagnosis of clinically relevant cancer [37]. Two notable disadvantages of the semi-quantitative parameters derived from DCE imaging is their direct estimation from the signal intensity measurements without physiological or empirical correlation and also their dependence on experimental factors such as sequence parameters or contrast dose, especially limiting their comparability and reproducibility between different sites [38].

In the present study, patients did not receive dynamic imaging, but only an early postcontrast phase. In everyday clinical practice, the time required for extensive examination protocols can be limited. Therefore, the objective of the present study was to test the performance and feasibility of a relatively short standard protocol for the preoperative evaluation of inferior vena cava wall invasion.

Regarding the results of the present study for MRI, the high sensitivity (92.3%) indicates, that the presence of IVC invasion is rarely underestimated and the high negative predictive value (90.5%) suggests, that if the IVC thrombus does not show any contact with the vessel wall, an IVC wall invasion can be reliably excluded. The comparably lower specificity shows, that a visual contact of the IVC thrombus with the vessel wall does not always correspond to IVC wall invasion, but still in more than 85% of the cases.

The excellent interobserver agreement for the assessment indicates the feasibility of using MR features for assessing IVC thrombus invasion in clinical practice. Complete occlusion of the IVC lumen or breach of the vessel wall may be used to predict the presence of IVC wall invasion and thus of complicated surgery.

This study has some limitations. Firstly, different MRI scanners were included over a relatively long period of time, resulting in a potential variability across scanners and imaging sessions, where the same scanner is used. Secondly, due to its retrospective design, a potential selection bias cannot be excluded. Thirdly, as intraoperative findings were used as the reference standard for wall invasion, presence of microscopic invasion cannot be excluded. Fourthly, as the present study is single center, external validation of the applied MR features is warranted. Furthermore, the addition of DWI or DCE imaging to the protocol might have further improved the preoperative assessment of IVC thrombus. Finally, MR imaging is contraindicated in some patients, e.g. patients with pacemakers, metallic foreign bodies or with severe claustrophobia.

## Conclusions

This study indicates that standard preoperative MR imaging can be used to reliably assess IVC wall invasion, evaluating morphologic features such as the complete occlusion of the IVC lumen or vessel breach. The excellent interobserver agreement suggests an adequate reproducibility of the preoperative assessment in clinical practice. In future, MR morphologic features might be used to refine preoperative planning and improve prior patient information.

## Abbreviations

CT: Computed tomography
GRE: Gradient-echo
ICC: Intraclass coefficient
IVC: Inferior vena cava
MRI: Magnetic resonance imaging
RCC: Renal cell carcinoma
